# Profiling of sesquiterpenoid fractions from *Artemisia annua* L. and testing their *in vitro* anti-SARS-CoV-2 activity

**DOI:** 10.17305/bb.2025.12052

**Published:** 2025-04-25

**Authors:** Irma Gušić, Ilma Terzić, Toni Eterović, Adis Softić, Šejla Goletić, Teufik Goletić, Dejan Nikolić, Emina Korić, Katarina Bijelić, Haris Nikšić, Senka Vidović, Kemal Durić

**Affiliations:** 1 Department of Pharmacognosy, Faculty of Pharmacy, University of Sarajevo, Sarajevo, Bosnia and Herzegovina; 2 Veterinary Faculty, University of Sarajevo, Sarajevo, Bosnia and Herzegovina; 3 Department of Pharmaceutical Sciences, Retzky College of Pharmacy, University of Illinois at Chicago, Chicago, IL, United States; 4 Department of Pharmacy, Faculty of Medicine, University of Novi Sad, Novi Sad, Serbia; 5 Center for Medical and Pharmaceutical Investigations and Quality Control, Faculty of Medicine, University of Novi Sad, Novi Sad, Serbia; 6 Department of Pharmaceutical Engineering, Faculty of Technology Novi Sad, University of Novi Sad, Novi Sad, Serbia

**Keywords:** Alpha variant SARS-CoV-2, antiviral activity, arteannuin B, *Artemisia annua L.*, countercurrent chromatography, supercritical fluid extraction

## Abstract

The current state of research on the anti-SARS-CoV-2 potential of artemisinin-related compounds has identified arteannuin B as a potent inhibitor of the nCoV-2019BetaCov/Wuhan/WiV04/2019 and BetaCov/Italy/CDG1/2020 strains of the virus. The aim of this work was to fractionate the targeted sesquiterpenoid compounds, arteannuin B and artemisinin, from the complex matrix of the crude ethanolic leaf extract of *Artemisia annua* L. using high-speed countercurrent chromatography (HSCCC) and to test the simplified or purified fractions against the genomically characterized Alpha SARS-CoV-2 variant *in vitro*. This is the first detailed *in vitro* anti-SARS-CoV-2 study using an analytically characterized supercritical fluid extract of *A. annua* L. The preparative HSCCC method enabled the isolation of purified arteannuin B in a single chromatographic step, which was confirmed by LC-ESI-QTOF-MS/MS. The MS data confirmed the selectivity of the HSCCC method for the targeted fractionation of artemisinin from the complex matrix, as it was successfully separated from the EtOH crude extract without co-elution with arteannuin B. Antiviral activity determined by quantitative real-time PCR (qRT-PCR) yielded half-maximal effective concentrations (EC_5__0_) of 93.7 µg/mL (SC-CO_2_ extract), 173.5 µg/mL (EtOH extract), 187.3 µg/mL (artemisinin knockout fraction), 38.1 µg/mL (arteannuin B fraction), and >100 µg/mL (artemisinin). The arteannuin B fraction was highly active at 50 µg/mL (*p* < 0.0001) and 100 µg/mL (*p* < 0.0001), and inhibited the amplification of the SARS-CoV-2 N and RdRp genes by 84% and 100%, respectively. An important contribution of this study is the demonstration of the antiviral activity of arteannuin B against the Alpha variant of SARS-CoV-2, which is known to have increased infectivity and transmissibility.

## Introduction

Severe acute respiratory syndrome coronavirus-2 (SARS-CoV-2) emerged as a major global health problem following the COVID-19 pandemic from 2020 to 2023. The global response to COVID-19 has provided valuable insights into the development and optimization of therapeutic strategies for viral infections. Significant progress was made during the pandemic in identifying effective antiviral interventions, such as monoclonal antibodies (mAbs) [1] targeting the outer spike protein of SARS-CoV-2, although mAbs were not effective against all circulating variants [2]. At the same time, the limitations of repurposed drugs, such as chloroquine, hydroxychloroquine, ivermectin, and lopinavir/ritonavir became clear [3, 4]. Repurposed drugs were attractive options as they were available for clinical use and had a favorable safety profile. However, their use is no longer recommended as there are no high-quality clinical trials demonstrating their antiviral benefits [5]. Novel direct-acting antiviral drugs, such as nirmatrelvir–ritonavir, which target the SARS-CoV-2 protease or polymerase, have been shown to be more durable than anti-SARS-CoV-2 mAbs for all SARS-CoV-2 variants. Other recommended antiviral therapies include intravenous remdesivir and oral alternative therapy with molnupiravir [6]. In the field of immunomodulators, while there is a relatively clear consensus on the value of corticosteroids for hospitalized patients requiring oxygen, there are still questions about the optimal timing and selection of a second immunomodulator, such as tocilizumab or baricitinib [7, 8]. Current antivirals have significant limitations, including drug–drug interactions (ritonavir-boosted nirmatrelvir), the need for intravenous administration (remdesivir) [9], and limited efficacy (molnupiravir), highlighting the need for new antiviral drugs without these shortcomings. Overall, the pandemic has highlighted the need to develop strategies for rapid drug repurposing and the development of targeted antivirals to prepare for future outbreaks.

Among natural products, *Artemisia annua* L. has established itself as an important source of anti-SARS-CoV-2 agents. This is underpinned by its importance during the previous SARS-CoV outbreak in 2002–2003, during which its bioactive compounds showed significant antiviral properties, and *A. annua* L. ranked second in efficacy among 200 tested herbal drugs [10]. *A. annua* L. is the herbal source of artemisinin, an endoperoxide-containing sesquiterpenoid with potent antimalarial activity [11, 12], as well as several artemisinin-related compounds and other natural products [13]. Owing to its medicinal properties [14], as evidenced by studies [15–17], many investigations have focused on identifying bioactive compounds other than artemisinin from *A. annua* L. The plant’s known antiviral efficacy [18–20], combined with its potential to modulate the immune response [21], underscores its value in the search for COVID-19 treatments.

In previous studies, ethanolic [22, 23] and hot water extracts [23–26] of *A. annua* L. showed *in vitro* activity against SARS-CoV-2. This activity was confirmed by plaque reduction assays [23], immunostaining for the SARS-CoV-2 spike glycoprotein [22], and the Renilla-Glo luminescence assay [24–26]. The anti-SARS-CoV-2 activity of extracts from *A. annua* L. was observed against various SARS-CoV-2 isolates, including SARS-CoV-2/human/Germany/BavPat 1/2020, SARS-CoV-2/human/Denmark/DK-AHH1/2020, and USA WA1/2020, as well as against variants of concern (VOCs), such as Alpha (B.1.1.7), Beta (B.1.351), Gamma (P.1), Delta (B.1.617), Kappa (B.1.617.1), and several Omicron sub-variants (BA.1, BA.2, BA.2.12.1, BA.4). Although artemisinin and dihydroartemisinic acid were detected in the extracts studied, the specific bioactive compounds responsible for the observed antiviral activity have not been identified [22–26].

The current state of research on the anti-SARS-CoV-2 potential of artemisinin-related compounds has identified arteannuin B as a potent inhibitor of the nCoV-2019BetaCov/Wuhan/WiV04/2019 and BetaCov/Italy/CDG1/2020 strains of the virus [27–29]. Arteannuin B exerts its inhibitory effect by forming a covalent bond with the cysteine residue in the active site of the main protease of SARS-CoV-2 (cysteine-145). This mechanism, which involves a thiol-Michael addition, explains the anti-SARS-CoV-2 and bioactive properties of arteannuin B [30]. Further research into natural products, with a focus on artemisinin and related compounds such as arteannuin B, holds significant potential for the development of novel SARS-CoV-2 therapies. Research priorities could include the development of cost-effective methods to isolate bioactive compounds from the complex matrix of *A. annua* L. to improve accessibility and scalability. Advanced techniques, such as supercritical extraction and highly selective chromatography, can optimize this process. Targeted *in vitro* evaluation of these compounds against emerging SARS-CoV-2 VOCs would be beneficial to assess their antiviral spectrum and adaptability to viral mutations. This approach could deepen our understanding of the antiviral properties of natural products and facilitate the development of effective therapies that can be adapted to viral evolution.

The novelty and advantage of this research over the literature are: (i) Targeted fractionation: we performed targeted fractionation of artemisinin and targeted fractionation and purification of arteannuin B from the complex plant matrix of crude *A. annua* L. EtOH leaf extract by preparative high-speed countercurrent chromatography (HSCCC), which was confirmed by liquid chromatography-electrospray ionization-quadrupole time-of-flight mass spectrometry (LC-ESI-QTOF-MS/MS); (ii) Selective sample preparation: comprehensive preparation of samples for *in vitro* anti-SARS-CoV-2 assessment, focusing on arteannuin B-enriched HSCCC fractions. These fractions were selectively separated and confirmed to be artemisinin-free by LC-ESI-QTOF-MS/MS; (iii) Antiviral evaluation of selected test samples: the *in vitro* antiviral evaluation was performed on samples tested against the genomically characterized Alpha variant (B.1.1.7 + Q.*; hCoV-19/Bosnia and Herzegovina/VFS-UNSA-LMGFI031/2021; GISAID accession ID: EPI_ISL_1016969), which was classified as VOC Alpha according to the World Health Organization (WHO) variant nomenclature [31, 32]; (iv) First report: this is the first detailed *in vitro* anti-SARS-CoV-2 study using an analytically characterized supercritical fluid extract of *A. annua* L.

## Materials and methods

### Extraction and fractionation methods

The leaves of *A. annua* L. were collected in June 2023 in Zenica, Bosnia and Herzegovina. The plant material was harvested during the vegetative phase, shortly before flowering. Species identification was confirmed by Dr. Haris Nikšić from the University of Sarajevo, Faculty of Pharmacy, Bosnia and Herzegovina (voucher specimen 116/23). The collected raw material was air-dried at 20–25 ^∘^C and stored in dark, dry conditions until analysis.

Dried, powdered leaves (30 g) were extracted with 96% (v/v) ethanol (EtOH) at 25 ^∘^C in an ultrasonic bath, using two 30-min ultrasonic cycles at a 1:20 herbal substance-to-solvent ratio. The EtOH extracts were centrifuged at 4000 rpm for 20 min at 4 ^∘^C and evaporated to dryness, yielding 4.3 g (14.3% yield).

Targeted fractions containing artemisinin and arteannuin B from the crude EtOH leaf extract of *A. annua* L. were obtained by HSCCC. HSCCC separations were performed using a CCC-1000 high-speed countercurrent chromatograph (Pharma Tech Research Co., Baltimore, MD, USA) equipped with a self-balancing three-coil centrifuge (320 mL total volume). The system was connected to a digital dual-piston pump (Shimadzu LC-10ATvp) for solvent delivery and a fraction collector with an autosampler (LAMBDA OMNICOLL, LAMBDA Instruments GmbH, Switzerland). A biphasic solvent system consisting of n-hexane, ethyl acetate, ethanol, and water in a volume ratio of 6:4:5:4 (v/v) [33] was freshly prepared before the experiments by mixing the solvents in a separatory funnel. This mixture was shaken vigorously to achieve phase separation. The upper and lower phases were then collected in separate containers and degassed in an ultrasonic bath for 20 min. The aqueous phase was chosen as the mobile phase, while the organic phase served as the stationary phase in a descending chromatography setup. First, the column was filled with the upper stationary phase at a flow rate of 9 mL/min. The mobile phase was then pumped through the stationary phase at a rate of 1.5 mL/min and a rotation speed of 800 rpm until hydrodynamic equilibrium was reached. The latter was determined by tracking a change in the refractive index in the graduated cylinder and the appearance of a clear mobile phase eluting at the tail outlet. Equilibrium was reached after approximately 42 min of continuous operation, as evidenced by constant flow rates and a stable ratio between the mobile and stationary phases, with no further displacement of the stationary phase observed in the effluent. A system pressure of 6 bar was reached during this process. The volume of the extruded stationary phase was then measured to determine the retention of the stationary phase (Sf) relative to the total capacity of the column. A total of 235 mg of EtOH leaf extract of *A. annua* L. was dissolved in 10 mL of a 50:50 (v/v) mixture of stationary and mobile phase and injected into the column after equilibration. The sample was injected into the column using a six-port high-pressure injection valve with a 10 mL loop. Elution was performed at a rotation speed of 800 rpm and a flow rate of 1.5 mL/min. The fractions were collected at eight-minute intervals over 12 h.

Supercritical fluid extraction (SFE) of *A. annua* L. leaves with supercritical carbon dioxide (SC-CO_2_) was performed using a laboratory-scale high-pressure extraction system (HPEP, NOVA, Effretikon, Switzerland) under the conditions described in our previous work [34]. The extraction was carried out at a pressure of 300 bar, a temperature of 40 ^∘^C, and a duration of 3 h, with a CO_2_ flow rate of 0.194 kg/h. The separator was kept at a pressure of 15 bar and a temperature of 23 ^∘^C during the entire process. The SC-CO_2_ extract was then collected in glass vials and stored at −20 ^∘^C until analysis.

### Selection and preparation of *A. annua* L. test samples for *in vitro* anti-SARS-CoV-2 evaluation

The selection of *A. annua* L. samples for *in vitro* testing of efficacy against the SARS-CoV-2 Alpha variant was based on the analysis of two target sesquiterpenoids: artemisinin and arteannuin B. These compounds were found in two different types of extracts from *A. annua* L.: the SC-CO_2_ extract and the EtOH extract, which required the simultaneous application of different analytical techniques for their analysis. Artemisinin in the crude extracts obtained with SC-CO_2_ and EtOH was quantified by high-performance liquid chromatography with diode-array detection (HPLC-DAD), while arteannuin B in the SC-CO_2_ extract was analyzed by gas chromatography–mass spectrometry (GC–MS). The initial inclusion criterion for testing the antiviral activity of the crude SC-CO_2_ and EtOH extracts was the presence of both target analytes in the analyzed samples.

After fractionation of the complex EtOH crude extract of *A. annua* L. into 85 fractions by HSCCC, the effluent and the purity of the fractions with respect to the target sesquiterpenoids were monitored by thin-layer chromatography (TLC). TLC analysis was performed on silica gel 60 F_2__5__4_ aluminum plates (Merck, Darmstadt, Germany) with a hexane/ethyl acetate (3:1, v/v) eluent. Artemisinin and arteannuin B were detected after derivatization with anisaldehyde–sulfuric acid reagent at 366 nm.

LC-ESI-QTOF-MS/MS analysis was performed to identify the target sesquiterpenoids in the EtOH crude extract and in selected HSCCC fractions after the results of TLC monitoring became available. Fractions confirmed to contain artemisinin by both TLC and LC-ESI-QTOF-MS/MS were pooled, the solvent was removed under reduced pressure, and they were designated as the artemisinin fraction. All fractions that eluted before the artemisinin fraction and were confirmed to be free of artemisinin by both TLC and LC-ESI-QTOF-MS/MS were pooled, the solvent was removed under reduced pressure, and they were designated as the artemisinin knock-out fraction. Fractions containing arteannuin B, as confirmed by TLC and LC-ESI-QTOF-MS/MS, were evaporated to dryness under reduced pressure and designated as the arteannuin B fraction.

The following crude extracts and fractions were tested for their *in vitro* anti-SARS-CoV-2 activity: (1) SC-CO_2_ extract; (2) EtOH crude extract; (3) Artemisinin knock-out fraction; (4) Arteannuin B fraction; (5) Artemisinin. DMSO was chosen as the solvent for the *in vitro* experiments, and the stock solutions were stored at −20 ^∘^C until analysis. All sample dilutions were prepared with cell culture medium prior to the experiments.

### LC-ESI-QTOF-MS/MS analysis of targeted sesquiterpenoids

Reversed-phase separations were performed using a Waters XBridge 2.1 × 50 mm C8 column (3.5 µm particle size) with a mobile phase consisting of 0.1% formic acid (solvent A) and acetonitrile (solvent B), and the following gradient: 15–100% B over 12 min at a flow rate of 0.2 mL/min. The column temperature was controlled at 40 ^∘^C.

Mass spectrometric data were acquired using a Waters SYNAPT hybrid quadrupole/time-of-flight mass spectrometer (Milford, MA, USA), operated in positive ion electrospray mode. Data were acquired at a resolution of 10,000 FWHM using Leu-enkephalin as a lock mass, which was introduced via a separate sprayer. Tandem mass spectra were acquired in data-dependent mode with a ramped collision energy of 6–50 eV and argon as the collision gas. The capillary voltage was set to 3.6 kV, while the cone voltage and the extraction cone voltage were set to 25 V and 4.0 V, respectively. The source and desolvation temperatures were maintained at 110 ^∘^C and 320 ^∘^C, respectively. The desolvation gas flow rate was 500 L/h. Data acquisition was performed using MassLynx software.

### Quantitative analysis of artemisinin by HPLC

The artemisinin content in the SC-CO_2_ and EtOH extracts was quantified using HPLC. The Thermo Scientific Vanquish Core HPLC instrument (Thermo Scientific, UK) with a diode array detector (DAD) was used for the HPLC analyses. The HPLC-UV-based determination of artemisinin was performed according to the method previously developed by Qian et al. [35], with some modifications, including the concentration range, mobile phase flow rate, and injection volume. Prior to analysis, artemisinin was converted to the highly UV-absorbing compound Q260 [36]. Chromatographic separation was performed on a thermostated Nucleosil C18 column (250 mm × 4.6 mm, 5 µm; Macherey Nagel) maintained at 30 ^∘^C. The mobile phase consisted of methanol (A), acetonitrile (B), and phosphate buffer (containing 0.9 mM Na_2_HPO_4_ and 3.6 mM NaH_2_PO_4_, adjusted to pH 7.76) (C). To improve separation efficiency, a gradient elution program was used as follows: 0 min: 10% A, 10% B, 80% C; 5 min: 35% A, 10% B, 45% C; 7 min: 35% A, 10% B, 45% C; 13 min: 80% A, 10% B, 10% C; 15 min: 10% A, 10% B, 80% C; and 20 min: 10% A, 10% B, 80% C. The flow rate was kept constant at 1 mL/min, and the injection volume was set to 50 µL. All quantitative analyses were performed using external standardization based on the peak areas obtained by DAD at 260 nm. In accordance with the guidelines of the International Conference on Harmonization (ICH, 2005), the limits of detection (LODs) and the limits of quantification (LOQs) were determined using the signal-to-noise ratio, as specified in the European Pharmacopeia (Ph. Eur. 11.0) [37, 38]. In addition, real samples were spiked at three concentration levels to verify the method in terms of precision and recovery.

A standard stock solution of artemisinin (Sigma-Aldrich) at a concentration of 0.5 mg/mL was prepared with 96% (v/v) EtOH. A 200 µL aliquot of this solution was transferred to a 10 mL volumetric flask and diluted to 10 mL with 96% EtOH. This mixture was then combined with 4 mL of 0.2% NaOH and heated at 50 ^∘^C for 1 h. After cooling, the solution was diluted to the mark with 0.08 M acetic acid to prepare a working standard solution. A series of calibration solutions with concentrations of 0.5, 1, 2.5, 3.75, and 5 µg/mL were prepared by dilution with 96% EtOH. Dried SC-CO_2_ and EtOH extracts of *A. annua* L. were reconstituted in 1 mL of 96% EtOH and processed as described for the standard stock solution.

Each sample was quantified in triplicate, and artemisinin content was expressed as the mean microgram equivalent of the standard per milligram of dry extract (DE) (µg/mg DE).

### GC–MS analysis of targeted sesquiterpenoids

Terpene components in dry SC–CO_2_ and EtOH extracts of *A. annua* L. were identified by GC-MS on an Agilent 7890B gas chromatograph coupled to a 5997 A mass selective detector (Agilent Technologies, Waldbronn, Germany). Separation was performed on an HP-5MS capillary column (30 m × 0.25 mm, 0.25 µm; Agilent Technologies). Samples were dissolved in hexane and injected in split mode (20:1) at an inlet temperature of 220 ^∘^C (1 µL). The oven temperature program was as follows: (i) initial temperature: 60 ^∘^C; (ii) first ramp: rise at 3 ^∘^C/min to 246 ^∘^C, with a hold time of 3 min; (iii) second ramp: rise at 10 ^∘^C/min to 280 ^∘^C, with a hold time of 5 min. Helium was used as the carrier gas (1 mL/min), and the temperature of the mass selective detector (MSD) transfer line was set to 285 ^∘^C. Mass spectral data were recorded in scan mode (*m/z* ═ 30–550). Compound identification was confirmed by comparing the mass spectra with those in the NIST14.L library using the Automated Mass Spectral Deconvolution and Identification System (AMDIS) [39]. The relative abundance of each component was expressed as a raw percentage of the total ion current (TIC).

### Cell culture

African green monkey kidney Vero E6 cells (Vero C1008, Vero 76, clone E6; ATCC CRL-1586™) were cultured and maintained in Dulbecco’s Modified Eagle Medium (DMEM-high glucose; Sigma-Aldrich), supplemented with 10% fetal bovine serum (FBS; Sigma-Aldrich), 2 mM L-glutamine (Sigma-Aldrich), and 1% antibiotic-antimycotic solution (Sigma-Aldrich). The cells were incubated at 37 ^∘^C in a humidified atmosphere with 5% CO_2_. They were passaged every three days with trypsin (Sigma-Aldrich) to maintain a subconfluent monolayer.

### Cell viability assay

The design of the cell viability assay was adapted to the experimental setup of the antiviral assays on Vero E6 cells. The Vero E6 cells were seeded at a density of 2 × 10^4^ cells per well in 96-well plates, with 100 µL of medium per well. The next day, the medium was replaced with 50 µL of medium containing extracts, fractions, or compounds; alternatively, 50 µL of medium with diluent (DMSO) was added. After a 90-min incubation at 37 ^∘^C and 5% CO_2_ in the presence of twofold serial dilutions of the test extracts, fractions, and compounds, 50 µL of fresh medium was added to each well, resulting in the indicated final concentrations of SC-CO_2_ and EtOH extracts (from 400 to 3.125 µg/mL), the artemisinin knockout fraction (from 400 to 3.125 µg/mL), the arteannuin B fraction, and artemisinin (from 200 to 1.5625 µg/mL). The treated cells were incubated for 48 h at 37 ^∘^C and 5% CO_2_, after which the culture supernatant was aspirated. Subsequently, 90 µL of fresh medium and 10 µL of MTT reagent (Sigma-Aldrich) were added to each well. The wells were incubated with MTT for 3 h. Then, 150 µL of DMSO was added to each well to dissolve the formazan crystals, followed by another incubation at 37 ^∘^C for 60 min. The optical density was measured at 570 nm using the BioTek Synergy™ LX multi-mode reader (Agilent Technologies, Santa Clara, USA). Untreated cells were designated as negative controls, and medium without cells served as a blank to evaluate the efficacy of the assay procedure [40]. The MTT assay was performed in triplicate. The absorbance values of the treated samples were adjusted relative to the blank and negative controls. Cell viability was determined according to (1):



(1)

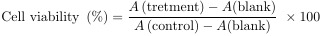




Normalized data were used for further statistical analysis.

### Genomic characterization of hCoV-19/Bosnia and Herzegovina/VFS-UNSA-LMGFI031/2021 by virus isolation and whole genome sequencing (WGS)

A nasopharyngeal swab (NPS) in a viral transport medium from a PCR-positive SARS-CoV-2 patient (RT-PCR COVID-19 positive male patient from Zenica, Federation of Bosnia and Herzegovina) from February 2021 was used for virus isolation. The virus isolate was obtained by inoculation of Vero E6 cells with the NPS and allowing virus propagation in Vero E6 cells at 90% confluence. Cytopathic effects (CPEs) were monitored daily in the infected cells using an inverted optical microscope (Olympus, Japan), and the virus was harvested when 80–90% of the cells manifested CPE. SARS-CoV-2 stocks were harvested 48 h post-infection (p.i.). The supernatant was collected, clarified, aliquoted, and stored at −80 ^∘^C for later use. RNA was extracted using the QIAamp Viral RNA Mini Kit (Qiagen, Germany), and the resulting product was subsequently used for further analysis. Amplification was performed using the LabGun COVID-19 ExoFast RT-PCR Kit (LabGenomics, Korea), according to the manufacturer’s instructions.

WGS was performed using the ARTIC amplicon sequencing protocol, optimized for MinION sequencing (Oxford Nanopore Technologies). Library preparation employed two distinct primer pools to generate overlapping amplicons spanning the entire viral genome [41]. Sequencing runs were performed on the MinION platform using an R9.4.1 flow cell, which produced high-quality reads with *Q* scores ≥7. Basecalling was performed using MinKNOW software, and consensus sequences were assembled by aligning raw reads to the Wuhan reference genome (GenBank ID: MN908947) using Minimap2, followed by error correction with Racon. The sequencing data were processed using the nCoV-2019 novel coronavirus bioinformatics pipeline [42]. Quality control metrics ensured that only high-fidelity reads were included. Variants were annotated using the Nextclade tool and Pangolin lineage assignment.

Virus propagation and all manipulations were performed in a biosafety level 3 facility at the University of Sarajevo, Faculty of Veterinary Medicine, Laboratory for Molecular-Genetic and Forensic Research.

The isolate hCoV-19/Bosnia and Herzegovina/VFS-UNSA-LMGFI031/2021 (GISAID accession ID: EPI_ISL_1016969) was used for further cell culture studies.

### Concentration-response antiviral assay in Vero E6 cells

The antiviral assays in Vero E6 cells were performed with some modifications according to established protocols for evaluating the antiviral activity of EtOH extracts from *A. annua* L. [22]. Vero E6 cells were seeded at a density of 2 × 10^4^ cells/well in 96-well plates and incubated overnight at 37 ^∘^C in a humidified 5% CO_2_ atmosphere. The following day, the medium was replaced with fresh medium containing the *A. annua* L. EtOH and SC-CO_2_ extracts, fractions, or compounds by adding 50 µL per well; alternatively, 50 µL of medium with diluent (DMSO) was added. After a 90-min incubation at 37 ^∘^C and 5% CO_2_ in the presence of twofold serial dilutions of the test extracts, fractions, and compounds, the cells were inoculated with hCoV-19/Bosnia and Herzegovina/VFS-UNSA-LMGFI031/2021 by adding 50 µL of the diluted virus stock per well, resulting in the indicated final concentrations of SC-CO_2_ and EtOH extracts (from 400 to 3.125 µg/mL), the artemisinin knockout fraction (from 400 to 3.125 µg/mL), the arteannuin B fraction and artemisinin (from 200 to 1.5625 µg/mL), and the diluent DMSO (0.5%), respectively. The virus dilution to be used as inoculum was selected together with the corresponding Ct value based on previous experiments (data not shown) to prevent virus-induced CPEs during the assay. Infected plates were left at 37 ^∘^C in a humidified atmosphere with 5% CO_2_. After 48 ± 1 hpi, the plates were frozen at −20^∘^C to allow virus load quantitation in the culture wells. After one cycle of freeze/thaw, each culture supernatant was collected for RNA extraction. Viral RNA extraction was performed using the Genolution NX-48S Viral NA Kit (VN143, Genolution Inc., Seoul, South Korea) on the Nextractor^®^ NX-48S Automated Extraction System (Genolution Inc., Seoul, South Korea). The extracted RNAs were analyzed using a TaqMan RT-PCR (LabGun COVID-19 ExoFast RT-PCR Kit) targeting the SARS-CoV-2 N gene (nucleocapsid) and RNA-dependent RNA polymerase (RdRp) gene, according to the manufacturer’s instructions. Thermal cycling was performed using the Applied Biosystems QuantStudio™ 5 Real-Time PCR System (Thermo Fisher Scientific, Waltham, MA, USA).

The concentrations were tested in quadruplicate, and the tests included 12 infected, untreated virus controls and 8 uninfected, untreated controls. An equivalent volume of solvent medium was administered to the control groups. The untreated virus controls were established as the reference point for the assessment of replication capacity within the experimental setup. The Ct values for the treated samples were adjusted both with respect to the virus control group and the initial virus titer of the virus dilution used as inoculum. This normalization allowed for an accurate calculation of the percentage inhibition of viral replication, which was then used for subsequent statistical analysis. The percentage of inhibition was calculated as follows (2):



(2)

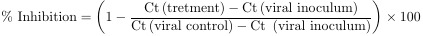




### Chemicals and reagents

Authentic artemisinin and arteannuin B standards for compound identification were obtained from Sigma-Aldrich and MedChemExpress. All solvents used for extraction and HSCCC fractionation were of analytical grade and sourced from Honeywell (Honeywell Research Chemicals). Solvents used for HPLC-DAD and LC-ESI-QTOF-MS/MS analyses were obtained from Fisher Scientific (Fair Lawn, NY, USA) and were at least HPLC grade. Carbon dioxide (99.9% w/w purity) was purchased from Messer, Novi Sad, Serbia, and used as the solvent for the SFE experiments in the laboratory.

### Ethical statement

The NPS of a patient was taken after obtaining the patient’s informed consent. Informed consent was an integral part of the questionnaire, which ensured that the participants understood and agreed to the use of anonymized data in accordance with ethical standards. This study was approved by the University of Sarajevo–Faculty of Pharmacy Ethical Review Committee (ERC# 2022-0101-3225) and the University of Sarajevo–Institute for Genetic Engineering and Biotechnology Ethical Review Committee (ERC# 2022-362).

### Statistical analysis

Statistical analyses were performed using Microsoft Excel 2016 and GraphPad Prism (version 10.2.3; GraphPad Software Inc., San Diego, CA, USA). Data are expressed as mean ± SD. EC_50_ and IC_50_ values were calculated using a nonlinear regression dose–response inhibition curve and plotted using GraphPad Prism software. The normality of the distribution of antiviral and MTT assay parameters was tested using the Shapiro–Wilk test. One-way analysis of variance (ANOVA) was then performed to analyze the results of the antiviral and MTT assays, followed by Dunnett’s test for multiple comparisons. In the case of a two-group comparison, the results were analyzed using a two-tailed Student’s *t*-test or Welch’s *t*-test. The significance level was set at *p < 0.05, **p < 0.01, ***p < 0.001, ****p < 0.0001.

## Results

### Identification of targeted sesquiterpenoids in *A. annua* L. fractions by LC-ESI-QTOF-MS/MS

**Figure 1.**
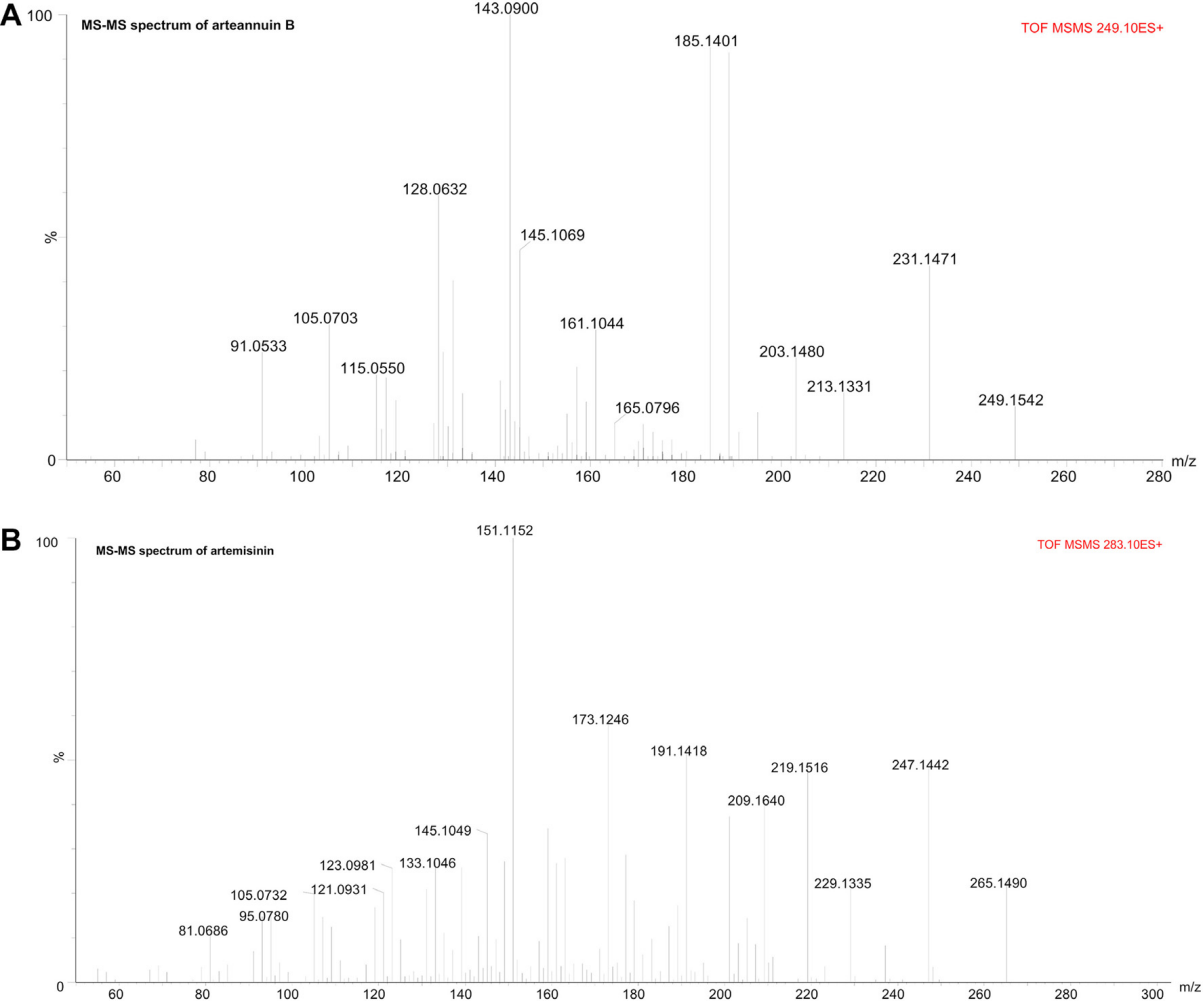
**Tandem mass spectra of arteannuin B and artemisinin acquired by ESI-QTOF-MS/MS in positive ion mode.** Representative MS/MS spectra used to confirm the identity of the target sesquiterpenoids detected in the *Artemisia annua* ethanolic extract and subsequent fractions. Panels show the characteristic fragmentation patterns of (A) arteannuin B with precursor ion [M+H]^+^ at m/z 249; and (B) Artemisinin with precursor ion [M+H]^+^ at m/z 283. The observed fragment ions match literature and reference standard data, supporting confident identification and the selectivity of the downstream HSCCC fractionation. MS/MS: Tandem mass spectrometry; ESI: Electrospray ionization; QTOF: Quadrupole time-of-flight; HSCCC: High-speed counter-current chromatography; m/z: Mass-to-charge ratio.

The identification of the targeted sesquiterpenoids, artemisinin and arteannuin B, in the test samples was performed by comparing the retention times and fragmentation patterns of the target compounds with those of the authentic standards. Detailed mass spectrometry data for arteannuin B and artemisinin can be found in Table 1, while the ESI-Q-TOF MS/MS spectra in positive ion mode are shown in Figure 1.

**Table 1:** Tandem mass spectrometry data for arteannuin B and artemisinin in test samples of *Artemisia annua* L., acquired using LC-ESI-QTOF-MS/MS

**Target compound**	**t_R_ (min)^1^**	**Formula**	**[M + H] ^+^ (*m/z*)**	**Production mass (*m/z*)**
Arteannuin B	8.49–8.63	C_15_H_20_O_3_	249.15	231.14
				189.09
				185.14
				145.10
				143.09
				128.06
Artemisinin	9.26–9.32	C_15_H_22_O_5_	283.16	265.15
				247.14
				219.15
				209.16
				191.14
				173.12
				151.11

^1^Retention time, min.

The MS spectrum of arteannuin B showed the protonated molecule [M+H]^+^ at *m/z* 249.15, along with the ammoniated and sodiated adducts at *m/z* 266.17 and 271.13, respectively. In addition, numerous in-source fragments were observed at *m/z* 231.14 [M+H–H_2_O]^+^, *m/z* 189.09 [M+H–C_3_H_8_O]^+^, and *m/z* 185.14 [M+H–2H_2_O–CO]^+^. The tandem mass spectrum of arteannuin B is shown in Figure 1.

**Figure 2.**
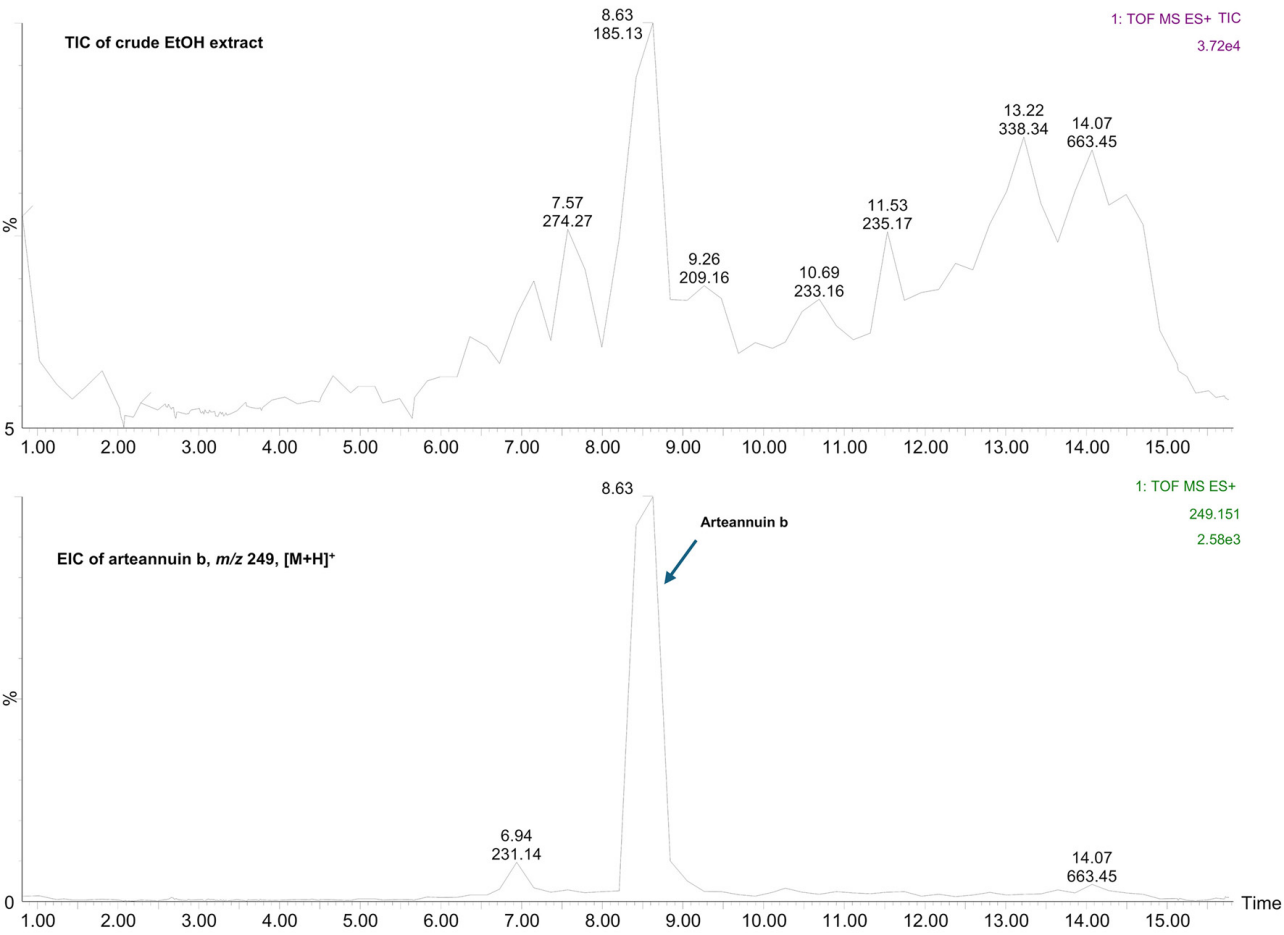
**TIC of the crude EtOH extract of *Artemisia annua* and EIC of arteannuin B.** The TIC profile of the crude ethanolic leaf extract (top) provides a molecular fingerprint of the complex mixture, highlighting multiple constituents. The EIC at m/z 249 [M+H]^+^ (bottom) confirms the presence of arteannuin B, detected as a distinct peak at 8.63 min. These results verify arteannuin B as one of the main compounds in the crude extract, thereby supporting its targeted isolation and further fractionation. TIC: Total ion chromatogram; EIC: Extracted ion chromatogram; EtOH: Ethanol; min: Retention time in minutes; m/z: Mass-to-charge ratio; [M+H]^+^: Protonated molecular ion.

**Figure 3.**
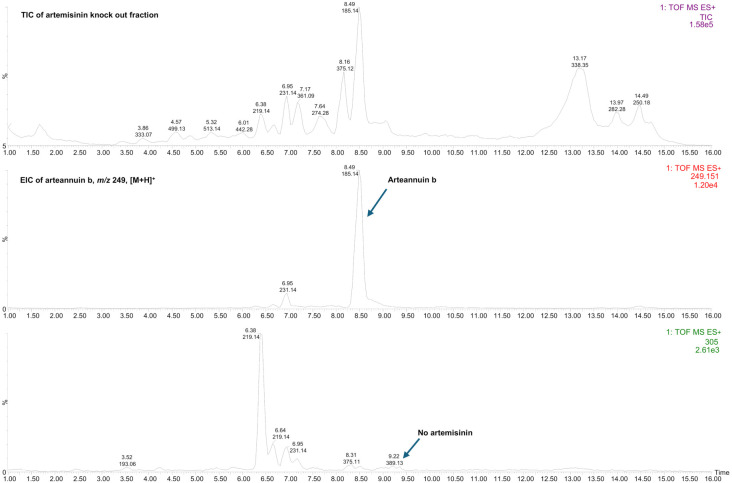
**TIC and EIC of the artemisinin knockout fraction of *Artemisia annua*.** The TIC (top) shows the overall chemical profile of the fraction. The EIC at m/z 249 [M+H]^+^ (middle) confirms arteannuin B at 8.48 min, while the EIC at m/z 305 [M+Na]^+^ (bottom) demonstrates the absence of artemisinin, confirming the selectivity of the fractionation. TIC: Total ion chromatogram; EIC: Extracted ion chromatogram; m/z: Mass-to-charge ratio; [M+H]^+^: Protonated molecular ion; [M+Na]^+^: Sodium adduct ion.

**Figure 4.**
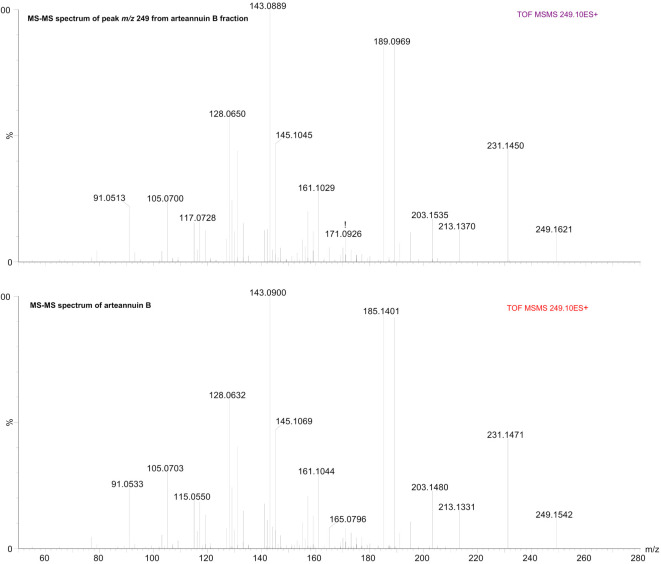
**Comparative ESI-QTOF-MS/MS spectra (positive ion mode) of arteannuin B: HSCCC fraction vs. reference standard.** Overlaid MS/MS spectra for the precursor ion m/z 249 [M+H]^+^ from the arteannuin B fraction (top) and the pure compound (bottom) show matching fragmentation patterns, confirming unambiguous identification and high purity of the isolated fraction obtained by HSCCC. ESI: Electrospray ionization; QTOF: Quadrupole time-of-flight; MS/MS: Tandem mass spectrometry; HSCCC: High-speed counter-current chromatography; m/z: Mass-to-charge ratio; [M+H]^+^: Protonated molecular ion.

The presence of arteannuin B was confirmed both in the crude EtOH extract and in the artemisinin knockout fraction (Figures 2 and 3). Purified arteannuin B was successfully isolated from the crude EtOH extract by HSCCC. The MS/MS spectrum of the arteannuin B fraction was identical to that of the pure compound (Figure 4). MS analysis confirmed the absence of arteannuin B in the artemisinin fraction, demonstrating that the chromatographic conditions and TLC monitoring of the analyte effectively ensured its proper separation and identification (Figure S1).

For artemisinin, the observed peaks in the MS spectrum were the protonated molecule [M+H]^+^ at *m/z* 283.16, the sodiated molecule at *m/z* 305.14, along with a series of in-source fragment ions at *m/z* 265.15 [M+H-H_2_O]^+^, *m/z* 247.14 [M+H-2H_2_O]^+^, *m/z* 219.15 [M+H-2H_2_O-CO]^+^, and *m/z* 209.16 [M+H-H_2_O-CO-C_2_H_4_]^+^. The tandem mass spectrum of artemisinin is shown in Figure 1.

The EIC of the sodiated molecule at *m/z* 305, extracted from the TIC of the crude EtOH extract, confirmed the presence of artemisinin in the analyzed sample (Figure S2). Artemisinin was neither detected in the artemisinin knockout fraction nor in the arteannuin B fraction (Figures 3 and S3). The MS spectrum of the artemisinin fraction confirmed the successful application of the HSCCC method to extract artemisinin from the complex EtOH crude extract in a single chromatographic step (Figure S1).

More detailed results are shown in Figure S3.

### Quantitative analysis of artemisinin

The HPLC-DAD method was successfully used to quantify artemisinin in the investigated *A. annua* L. SC-CO_2_ and EtOH extracts. Figure S4 shows the HPLC-DAD chromatograms for the blank solvent, the working standard solution, and representative *A. annua* L. samples. The chromatograms confirm the successful conversion of artemisinin to the highly UV-absorbing compound Q260, as evidenced by a peak eluting at 5.23 min. No significant interference with the analyte peak was observed, indicating high selectivity of the experimental method used.

Good linearity was observed for artemisinin in the range of 0.5–5 µg/mL, with a regression equation of *y* ═ 0.0213*x* −0.3293 and a correlation coefficient of R^2^ ═ 0.9995. The LOD and LOQ were 0.2 µg/mL and 0.5 µg/mL, respectively. The precision was 1.4% (RSD), and the recovery ranged from 98% to 102%.

Table 2 shows the artemisinin content (in µg/mg DE ± SD) in the extracts of *A. annua* L. obtained by two different extraction methods. SC-CO_2_ extraction resulted in a significantly higher artemisinin content (*p* < 0.0001) compared to ultrasonic extraction with EtOH, with values of 14.65 ± 0.28 µg/mg and 6.86 ± 0.39 µg/mg, respectively.

**Table 2:** Content of artemisinin in test samples of *Artemisia annua* L. determined by HPLC-DAD

**No.**	**Type of extract**	**Artemisinin content (µg/mg DE), mean ± SD**	**Extraction method**
S-01	SC-CO_2_ extract	14.65 ± 0.28****	Supercritical carbon dioxide extraction
S-02	EtOH extract	6.87 ± 0.39	Ultrasonication

****significance level *P* < 0.0001. SD: Standard deviation; DE: Dry extract.

### GC–MS analysis of targeted sesquiterpenoids

The detailed chemical compositions of the *A. annua* L. SC-CO_2_ and EtOH extracts, determined by GC–MS analysis, are listed in Table 3. A total of 24 compounds were identified, of which 23 compounds accounted for 90.16% of the SC-CO_2_ extract, while 15 compounds accounted for 89.25% of the EtOH extract, based on the TIC. Oxygenated sesquiterpenes (43.82% in the SC-CO_2_ extract and 52.84% in the EtOH extract) were the most abundant compound class in both samples analyzed.

The major constituents (>2.0%) in the SC-CO_2_ extract of *A. annua* L. included the oxygenated monoterpene endo-borneol (14.56%), the sesquiterpene β-selinene (16.72%), and the oxygenated sesquiterpenes deoxyartemisinin (2.1%), β-cyclocostunolide (2.75%), longicamphenylone (3.88%), caryophyllene oxide (4.05%), costunolide (6.27%), and arteannuin B (18.95%). The diterpenoid phytol was also present at 2.41%, and durohydroquinone at 3.05%. Monoterpenoids accounted for 21.85% of the total compounds identifnoids for 58.2%, diterpenoids for 2.41%, and other compounds for 3.05%.

The qualitative content of non-polar components (>2.0%) in the EtOH extract of *A. annua* L. included the oxygenated monoterpenes (+)-camphor (2.43%) and endo-borneol (15.49%), the sesquiterpene β-selinene (14.90%), and the oxygenated sesquiterpenes (1R,7S)-Germacra-4(15),5,10(14)-trien-1β-ol (2.19%), deoxyartemisinin (2.5%), longicamphenylone (2.68%), caryophyllene oxide (4.21%), arteannuic acid (4.92%), costunolide (5.11%), and arteannuin B (27.18%). Monoterpenoids accounted for 18.47% of the total compounds identified, and sesquiterpenoids for 61.62%.

### Genomic characterization of the viral isolate

The genome analysis of hCoV-19/Bosnia and Herzegovina/VFS-UNSA-LMGFI031/2021 (GISAID accession ID: EPI_ISL_1016969) revealed that the viral genome consists of 29,903 nucleotides, which is consistent with canonical SARS-CoV-2 genomes. The analysis identified the following key mutations in the isolate hCoV-19/Bosnia and Herzegovina/VFS-UNSA-LMGFI031/2021: (i) PLpro: T183I, A890D, I1412T; (ii) nsp6: Δ106-108; (iii) RdRP: P323L; (iv) Spike: Δ69-70, Δ144, N501Y, A570D, D614G, P681H, T716I, S982A, D1118H; (v) ORF8: Q27*, R52I, K68*, Y73C; (vi) N: D3L, R203K, G204R, S235F.

The Nextclade tool and Pangolin lineage assignment identified the isolate as belonging to the B.1.1.7 lineage (Pango v4.3.1 consensus call), classified as Alpha (B.1.1.7-like), and formerly designated as VOC Alpha GRY (B.1.1.7 + Q.*) according to the WHO variant nomenclature [31, 32].

These mutations indicate alterations in structural and non-structural proteins that affect viral infectivity, replication and evasion of the immune response.

### Cell viability assay

**Figure 5.**
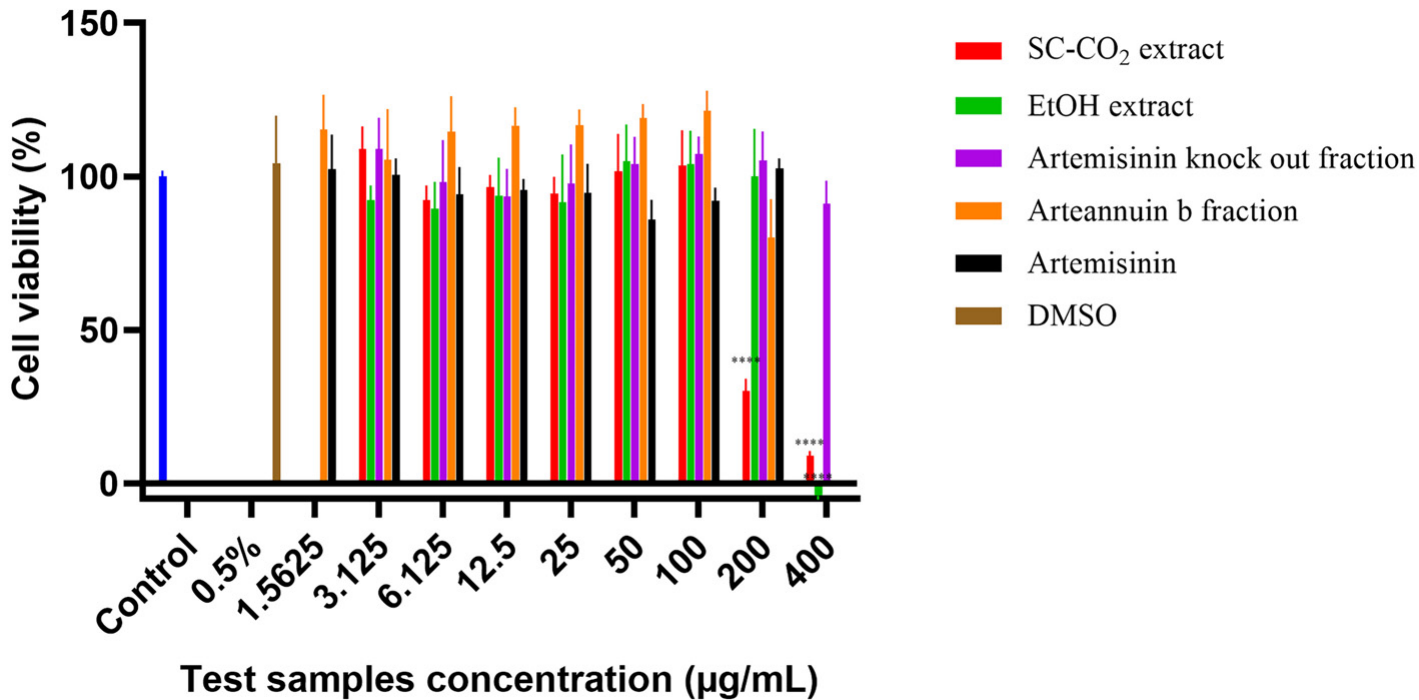
**Normalized cell viability (%) measured by MTT assay after exposure of Vero E6 cell monolayers to different concentrations of each tested *A. annua* L. sample for 48 h.** Data are expressed as the mean of the three independent replicates ± standard deviations. Statistically significant differences compared to controls (*p < 0.05, **p < 0.01, ***p < 0.001, ****p < 0.0001).

The MTT assay was performed to evaluate the viability of Vero E6 cells after treatment with the *A. annua* L. test samples. The results of cell viability of Vero E6 cells in the presence of the tested crude extracts, fractions, and compounds of *A. annua* L. are shown in Figure 5. The cell viability assays yielded half-maximal inhibitory concentrations (IC_5__0_) of 191.8 µg/mL (SC-CO_2_ extract), 239.1 µg/mL (EtOH extract), >400 µg/mL (artemisinin knockout fraction), >200 µg/mL (arteannuin B fraction), and >200 µg/mL (artemisinin). Test samples were diluted in DMSO to a final concentration of 0.5%, which did not independently reduce cell viability (≥99%). No significant differences were observed in the mean values of percent cell viability between the control group and the 0.5% DMSO group (*p* > 0.05).

**Table 3:** Qualitative analysis of the non-polar constituents in SC-CO_2_ and EtOH extracts of *Artemisia annua* L. by GC–MS (% TIC)

**Peak no.**	**Compound**	**t_R_ (min)^1^**	**SC-CO_2_ extract**	**EtOH extract**
Monoterpene hydrocarbons			0.17	-
1	Camphene	7.97	0.17	n.d.
Oxygenated monoterpenes			21.68	18.47
2	1,8-Cineole	10.53	1.65	n.d.
3	Artemisia ketone	11.58	4.03	0.55
4	Artemisia alcohol	12.47	0.2	n.d.
5	(+)-Camphor	15.00	1.24	2.43
6	Endo-Borneol	15.96	14.56	15.49
Sesquiterpene hydrocarbons			19.03	17.94
7	α-Copaen	24.69	0.91	1.33
8	Caryophyllene	26.49	0.8	1.71
9	Amorpha-4,11-diene	28.70	0.6	n.d.
10	β-selinene	29.14	16.72	14.9
Oxygenated sesquiterpenes			43.82	52.84
11	Caryophyllene oxide	32.87	4.05	4.21
12	δ-Cadinol	34.73	1.45	n.d.
13	α-Bisabolol	36.50	0.45	n.d.
14	(1R,7S)-Germacra-4(15),5,10(14)-trien-1β-ol	36.65	0.93	2.19
15	Cis-14-normuurol-5-en-4-ol	39.46	1.49	n.d.
16	Longicamphenylone	39.78	3.88	2.68
17	Costunolide	43.40	6.27	5.11
18	Arteannuic acid	44.17	1.5	4.92
19	β-Cyclocostunolide	46.65	2.75	1.55
20	Velleral	47.14	n.d.	2.5
21	Deoxyartemisinin	48.18	2.1	2.5
22	Arteannuin B	48.94	18.95	27.18
Diterpenes			2.41	–
24	Phytol	50.50	2.41	n.d.
*Other compounds*				
23	Durohydroquinone	49.99	3.05	n.d.

^1^Retention time, min: n.d.: Non detected; % TIC: The relative abundance of each component was expressed as a raw percentage of the total ion current.

ANOVA revealed a significant difference in cell viability between the treatments administered (*p* < 0.0001). The post-hoc Dunnett’s multiple comparisons test showed statistically significant differences between the negative control and the treatments with SC-CO_2_ and EtOH extracts. These differences were observed only at the highest concentrations tested: 200 µg/mL and 400 µg/mL for the SC-CO_2_ extract, and 400 µg/mL for the EtOH extract. At lower concentrations of all treatments tested, no significant differences were observed compared to the negative control.

### Concentration-response antiviral assay in Vero E6 cells

**Figure 6.**
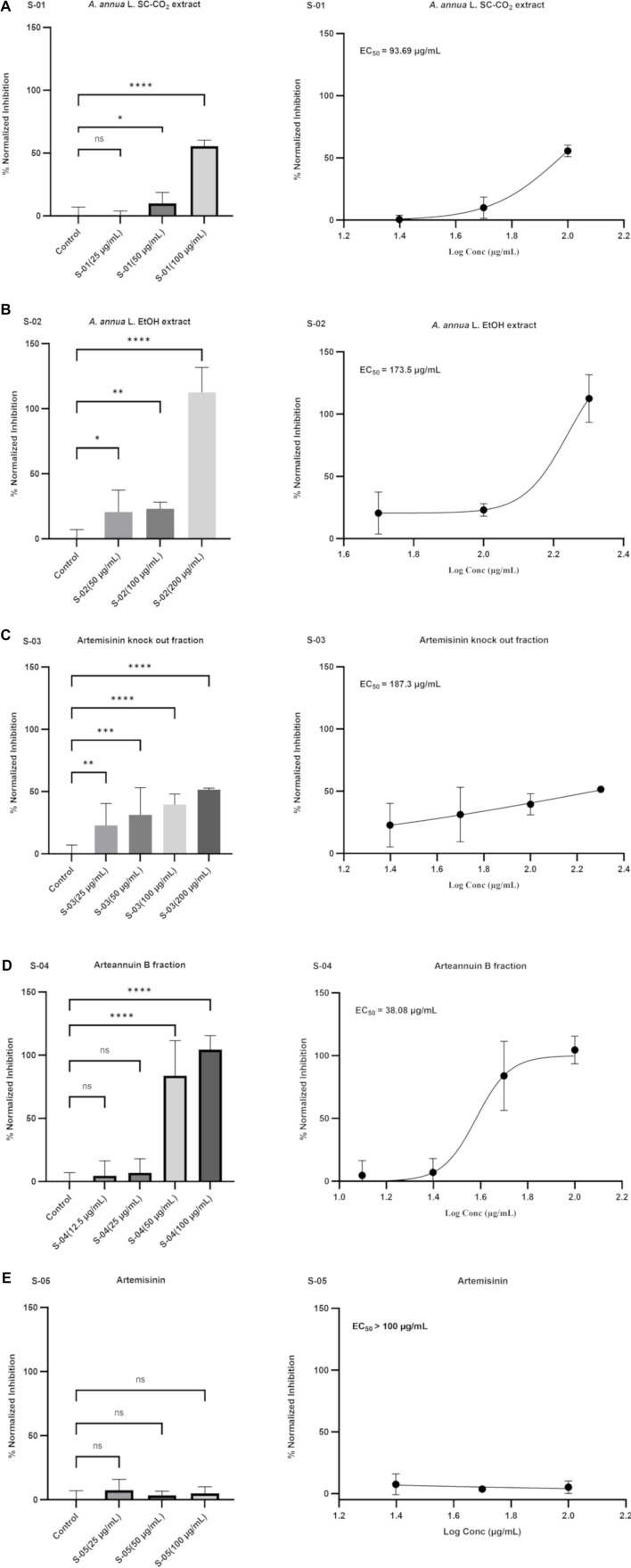
Normalized inhibition (%) of the Alpha variant (B.1.1.7 + Q.*; hCoV-19/Bosnia and Herzegovina/VFS-UNSA-LMGFI031/2021; GISAID accession ID: EPI_ISL_1016969) determined by quantitative real-time PCR after exposure of Vero E6 infected monolayers to different concentrations of each tested *Artemisia annua* L. sample for 48 h. Samples (A) *A. annua* L. SC-CO_2_ extract; (B) *A. annua* L. EtOH extract; (C) Artemisinin knockout fraction; (D) Arteannuin B fraction; (E) Artemisinin. Data are presented as means of independent replicates ± standard deviations. Statistically significant differences compared to controls (*p < 0.05, **p < 0.01, ***p < 0.001, ****p < 0.0001).

Experiments with crude extracts, fractions, and compounds of *A. annua* L. tested against the alpha variant (B.1.1.7 + Q.*; hCoV-19/Bosnia and Herzegovina/VFS-UNSA-LMGFI031/2021; GISAID accession ID: EPI_ISL_1016969) showed a clear concentration-dependent inhibition of viral RNA copies in the cell supernatant, as determined by quantitative real-time PCR (qRT-PCR). The antiviral activity resulted in half-maximal effective concentrations (EC_5__0_) of 93.7 µg/mL (SC-CO_2_ extract), 173.5 µg/mL (EtOH extract), 187.3 µg/mL (artemisinin knock-out fraction), 38.1 µg/mL (arteannuin B fraction), and >100 µg/mL (artemisinin) (Figure 6).

The highest concentrations evaluated in our antiviral assays were determined based on the results of the MTT assay to ensure that only concentrations maintaining cell viability above 85% were included in the evaluation (Figure 5). Figure 6 summarizes the antiviral effect, expressed as the percentage of virus inhibition relative to the tested concentrations of the *A. annua* L. test samples, along with the indicated levels of statistical significance: (A) the SC-CO_2_ extract exerted an antiviral effect when used at concentrations of 50 µg/mL (*p* < 0.05) and 100 µg/mL (*p* < 0.0001); (B) the EtOH crude extract retained considerable activity at all concentrations evaluated 50 µg/mL (*p* < 0.05) and 100 µg/mL (*p* < 0.01), while complete viral inhibition was achieved at the highest concentration tested, 200 µg/mL (*p* < 0.0001); (C) the artemisinin knockout fraction showed activity at 25 µg/mL (*p* < 0.01), with significance increasing at higher concentrations tested; (D) the arteannuin B fraction was highly active at 50 µg/mL (*p* < 0.0001) and 100 µg/mL (*p* < 0.0001) and inhibited the amplification of SARS-CoV-2 N and RdRp genes by 84% and 100%, respectively; (E) artemisinin showed no significant activity at the tested concentrations compared to the control group.

The test samples were diluted in DMSO to a final concentration of 0.5%, which did not independently reduce virus replication. No significant differences were found in the mean percentage values of virus replication between the control group and the group with 0.5% DMSO (*p* > 0.05).

## Discussion

The aim of this work was to fractionate the targeted sesquiterpenoid compounds, arteannuin B and artemisinin, from the complex matrix of crude *A. annua* L. EtOH leaf extract and to test the simplified or purified targeted fractions against the Alpha SARS-CoV-2 variant *in vitro*. In addition, an advanced extraction method using supercritical CO_2_ as an extraction solvent was employed to extract the relatively non-polar, thermolabile compounds, including arteannuin B and artemisinin, from a solid *A. annua* L. leaf matrix and to test the anti-SARS-CoV-2 activity of the obtained SC-CO_2_ extract *in vitro*. The first inclusion criterion for testing the crude SC-CO_2_ and EtOH extracts for antiviral activity was the presence of both target analytes in the analyzed samples (Tables 2 and 3, Figure 2 and Figure S2). Furthermore, it was important to confirm the presence of both analytes in the EtOH extract to ensure that the planned HSCCC fractionation could be successfully performed. This approach implied the use of a comprehensive analytical strategy.

The TIC chromatogram of the crude *A. annua* L. EtOH leaf extract obtained by MS detection is shown in Figure 2. It provides a molecular fingerprint of the sample, highlighting its complexity and revealing the fingerprints of arteannuin B as one of the main compounds. Separation and purification of arteannuin B by conventional methods, such as column liquid chromatography, require several steps, resulting in lower recovery [43], or the need for pre-fractionation of the crude extract prior to separation of the compound by preparative HPLC [27]. The preparative HSCCC method proposed here allowed the isolation of purified arteannuin B from a 235 mg extract in a single chromatographic step (Figure 4 and Figure S3). Moreover, no pre-purification of the extract prior to isolation or post-column purification of the target compound was required, as confirmed by LC-ESI-QTOF-MS/MS (Figure 4 and Figure S3). The proposed HSCCC method was proven to be suitable for the fractionation of artemisinin from the EtOH crude extract (Figure S1), while artemisinin could not be detected in either the artemisinin knockout fraction or the arteannuin B fraction (Figures 3 and 4). MS data confirmed these statements, indicating the good selectivity of the HSCCC method for targeted fractionation of artemisinin from the complex matrix. An LC system coupled to a Q-TOF mass spectrometer was used as an advanced tool for chemical profiling of the EtOH extract of *A. annua* L. and selected HSCCC fractions, allowing the simultaneous identification of arteannuin B and artemisinin. The high-resolution and rapid LC-ESI-QTOF-MS/MS method used provided mass spectrometry data, secondary mass spectra, and chemical structure fragmentation information consistent with data published in the literature [44–46]. Additional confidence in confirming the presence or absence of targeted sesquiterpenoids in the analyzed samples was achieved by comparing the tandem mass spectrometry data with reference standards to avoid false positive results.

The use of a green solvent in the preparation of the crude extract instead of highly toxic organic solvents [43], the subsequent efficient extraction of targeted sesquiterpenoids with ultrasound-assisted extraction, and the combination of several advantages of the applied fractionation technique (HSCCC)—such as high loading capacity, good selectivity, low solvent consumption, and the absence of sample loss due to irreversible adsorption or degradation within the column [47–49]—resulted in an approach that is both effective and significant for the production of *A. annua* L. fractions with high antiviral activity (Figure 6).

The efficiency of two extraction methods, SFE (SC-CO_2_) and ultrasonic extraction with EtOH for the extraction of artemisinin from *A. annua* L. was also investigated. Artemisinin, a thermally unstable compound, cannot be analyzed at temperatures above 180 ^∘^C without degradation by GC, unlike other sesquiterpenoids considered in this work [50, 51]. Instead, an HPLC-DAD method was used for the quantification of artemisinin, in which artemisinin is converted into the highly UV-absorbing compound Q260 [35]. Quantitative analysis revealed that the peak area of artemisinin obtained with SC-CO_2_ was significantly higher than that obtained with ultrasound-assisted extraction (*p* < 0.0001). Consequently, SC-CO_2_ was selected as the preferred method for artemisinin extraction and prioritized for further research. Thus, the current research results are consistent with published studies [52, 53].

GC–MS analysis (Table 3) of SC-CO_2_ and EtOH extracts of *A. annua* L. reveals a variety of compounds, including acyclic, monocyclic, and bicyclic monoterpenoids; monocyclic, bicyclic, and tricyclic sesquiterpenoids and various other chemical constituents. Acyclic monoterpenoids were represented by artemisia ketone in both the SC-CO_2_ and EtOH extracts, while artemisia alcohol was only present in the SC-CO_2_ extract. In addition, the acyclic diterpene alcohol phytol was only detected in the SC-CO_2_ extract. The monocyclic monoterpenoid 1,8-cineole was detected in the SC-CO_2_ extract. The group of bicyclic monoterpenoids was represented by camphene, which was found exclusively in the SC-CO_2_ extract, while (+)-camphor and endo-borneol were present in all tested extracts. Among the monoterpenoids, endo-borneol stands out due to its high content in both samples analyzed, while sesquiterpenoids were the predominant component in all samples analyzed and exceeded the content of monoterpenoids. Monocyclic sesquiterpenoids were represented by the presence of the germacrane sesquiterpenoid costunolide, β-cyclocostunolide, and (1R,7S)-Germacra-4(15),5,10(14)-trien-1β-ol in both extracts, whereas the bisabolane sesquiterpene α-bisabolol was only detected in the SC-CO_2_ extract. Bicyclic caryophyllane sesquiterpenoids were represented by caryophyllene and caryophyllene oxide in the analyzed samples. The three most abundant compounds in the samples included β-selinene, the most abundant bicyclic eudesmane sesquiterpene in *A. annua* L. [13]. The amorphane/cadinane group of bicyclic sesquiterpenes was the predominant class of sesquiterpenes in the *A. annua* L. samples. Cadinane and muurolane sesquiterpenoids were represented by the bicyclic compounds δ-cadinol and cis-14-normuurol-5-en-4-one, both of which were found exclusively in the SC-CO_2_ extract. Several bicyclic amorphane sesquiterpenes listed in Table 3 have been identified as biosynthetic precursors of artemisinin [54]. Most notably, amorpha-4,11-diene was detected in the SC-CO_2_ extract, while arteannuin acid and arteannuin B were present in both samples analyzed. Arteannuin B, a bicyclic amorphane sesquiterpene lactone, was the most abundant constituent in both extracts (based on TIC), with the highest concentration observed in the EtOH crude extract (27.18%). Deoxyartemisinin, which is considered a metabolite of dihydroartemisinic acid, was detected together with longicamphenylone, an oxygenated sesquiterpene, in both samples analyzed. Tricyclic sesquiterpenoids were represented by the presence of the copaene sesquiterpenoid α-copaene. In addition to the compounds listed above, the following were detected in the extracts of *A. annua* L. using the GC–MS method: velleral and durohydroquinone. We conclude that SFE is the preferred method for the extraction of sesquiterpenes from the leaves of *A. annua* L., from a qualitative point of view, compared to ultrasonic extraction with EtOH.

In accordance with the aim of this work, the antiviral potential of the test samples of *A. annua* L. against the Alpha variant of SARS-CoV-2 *in vitro* was evaluated after comprehensive analytical characterization and profiling of the crude extracts, fractions, and compounds, together with genome analysis of the local virus isolate. The use of the local isolate hCoV-19/Bosnia and Herzegovina/VFS-UNSA-LMGFI031/2021 offered a significant advantage and provided specific insights into the epidemiological situation in the region. This underscores the importance of monitoring viral evolution within localized populations to develop tailored therapeutic approaches and improve public health strategies. The Ct value range of 18–26 (as the initial Ct value of the virus used), observed in this study and consistent with previous observations, correlates with clinically manifest COVID-19 cases, emphasizing its importance for the assessment of viral replication and inhibition. In addition, the sensitivity of the PCR method used allowed reliable detection of viral load even at low concentrations, enabling accurate assessment of viral inhibition and the efficacy of treatments tested at different concentrations.

Genome analysis of hCoV-19/Bosnia and Herzegovina/VFS-UNSA-LMGFI031/2021 identified several critical features of the virus that provide insights into its genetic makeup and potential impact on infectivity and transmissibility. Among the mutations in the structural genes, the S (spike) protein mutation D614G was identified as a prominent alteration known to enhance viral infectivity by stabilizing the prefusion conformation of the spike trimer [55]. The mutation was globally prevalent, as it is associated with increased transmissibility. The Alpha VOC (B.1.1.7) [32] carried a combination of significant mutations in the S (spike) protein that influence its biological behavior and phenotypical features. The key mutations identified include Δ69-70, Δ144, N501Y, A570D, P681H, T716I, S982A, and D1118H. These mutations collectively enhance the transmissibility of the Alpha variant and alter its interaction with the host immune system. Deletions Δ69-70 and Δ144 were located in the N-terminal domain of the S protein and were associated with immune evasion by affecting antibody recognition, particularly mAbs targeting this region. The amino acid substitution N501Y occurred in the receptor-binding domain (RBD) of the S protein, increasing its binding affinity to the human angiotensin-converting enzyme 2 (ACE2) receptor [56, 57]. This heightened binding efficiency was a critical factor in the enhanced transmissibility of the Alpha variant. The A570D mutation, located adjacent to the RBD, is thought to stabilize the spike protein structure, further facilitating efficient ACE2 binding [58]. P681H, found near the furin cleavage site, enhanced the cleavage of the S protein, improving viral entry into human cells, which was considered a hallmark of increased infectivity [59]. T716I, S982A, and D1118H mutations were situated in the S2 subunit and contributed to structural changes that enhance the stability and functionality of the spike protein, aiding in viral fusion and entry [60]. In addition to these spike protein mutations, changes in the nucleocapsid (N) gene, such as D3L, R203K, G204R, and S235F, have also been observed [61]. These mutations might influence the efficiency of viral RNA packaging and replication, thereby potentially increasing the overall fitness of the virus. The R203K and G204R linked mutations were located in the serine/arginine (SR)-rich region of the nucleocapsid protein, which plays a crucial role in RNA binding and genome packaging [62]. D3L and S235F mutations might contribute to structural adjustments in the nucleocapsid protein, impacting viral stability and replication. Given the central role of the N protein in modulating viral transcription, such alterations could also affect the host immune response. Alterations in non-structural proteins, particularly those encoded by ORF1b, include the P323L (Orf1b: P314L) mutation in RdRp. Variants in accessory proteins encoded by ORF8 (Q27*, R52I, K68*, Y73C) have also been detected, with implications for host immune evasion and apoptosis. These mutations may reflect viral adaptations to evade host antiviral defenses and underscore the dynamic nature of SARS-CoV-2 evolution.

Evaluation of the effect of different concentrations of crude extracts, fractions, and compounds of *A. annua* L. on the viability of Vero E6 cells was performed using the MTT assay. The IC_50_ values for the SC-CO_2_ and EtOH extracts (191.8 µg/mL and 239.1 µg/mL, respectively) indicate that both extracts have a moderate inhibitory effect on the viability of Vero E6 cells. In contrast, the IC_50_ values of the artemisinin knock-out fraction, the arteannuin B fraction, and artemisinin were above 200 µg/mL, indicating a lower cytotoxic potential on the Vero E6 cells at the concentrations tested. None of the treatments below 200 µg/mL showed a significant difference in cell viability compared to the negative control, indicating that these concentrations had no effect on cell viability. Only the treatments with the highest tested concentrations of 200 µg/mL and 400 µg/mL for the SC-CO_2_ extract, and 400 µg/mL for the EtOH crude extract, were significantly different from the negative control (Figure 5), and these concentrations of SC-CO_2_ and EtOH samples were excluded from the antiviral evaluation. The results confirmed that concentrations up to 0.5% DMSO (used to dilute the samples) did not significantly affect cell viability, as no statistically significant differences were observed between the control group and the 0.5% DMSO group (*p* > 0.05). Therefore, it is unlikely that the significantly reduced cell viability observed when using the EtOH and SC-CO_2_ extracts at relatively high concentrations was due to DMSO (Figure 5).

Based on the results of the MTT assay, the antiviral potential of the *A. annua* L. test samples was evaluated in terms of cell viability and cytotoxicity at concentrations maintaining cell viability above 85%. The results presented here provide convincing evidence for the antiviral activity of the *A. annua* L. test samples against the Alpha variant of SARS-CoV-2, as determined by qRT-PCR. Our study shows a clear concentration-dependent inhibition of viral RNA replication in the cell supernatant, confirming the potential of *A. annua* L. extracts and fractions to attenuate viral replication. The SC-CO_2_ extract of *A. annua* L. significantly inhibited viral replication (EC_5__0_ of 93.7 µg/mL), with a notable effect observed at 100 µg/mL, where strong viral inhibition was achieved (*p* < 0.0001) (Figure 6). The data represent the first detailed *in vitro* anti-SARS-CoV-2 study using an analytically characterized supercritical fluid extract of *A. annua* L. The chemical composition of the SC-CO_2_ extract supports its potential as an effective antiviral agent against SARS-CoV-2. The extract is rich in oxygenated sesquiterpenoids (33.31%) (Table 3). In particular, compounds, such as arteannuic acid [27], caryophyllene oxide [63, 64], and arteannuin B [27–30] are known for their bioactive properties, including antiviral activity. The SC-CO_2_ extract also contains the monoterpenes and sesquiterpenes endo-borneol (14.56%) and β-selinene (16.72%) (Table 3). These compounds, especially endo-borneol, are thought to have various biological activities, including antimicrobial and antiviral properties [65–67]. The presence of these compounds in high concentrations in the extract could enhance its overall antiviral potential. The EtOH crude extract retained considerable activity at all concentrations tested (EC_5__0_ of 173.5 µg/mL), with complete viral inhibition achieved at 200 µg/mL (*p* < 0.0001) (Figure 6). These results are consistent with previous studies demonstrating the antiviral potential of *A. annua* L. extracts [22, 23, 27]. Among the biologically active constituents confirmed in this extract, arteannuin B stands out as one of the most abundant, based on the % TIC (Table 3, Figure 2), and may play a key role in contributing to the antiviral potential of the extract. The artemisinin knockout fraction showed antiviral activity at lower concentrations, with significant inhibition observed at 25 µg/mL (*p* < 0.01), which increased with higher concentrations (Figure 6). MS analysis confirmed the presence of arteannuin B in the artemisinin knockout fraction and, at the same time, the absence of artemisinin (Figure 3).

The presented antiviral results for the artemisinin knockout fraction and the unique design of the sample preparation provide direct evidence that bioactive compounds other than artemisinin in the tested artemisinin knockout fraction may contribute to the observed antiviral effects. As previously shown [26], there was an inverse correlation between artemisinin and antiviral efficacy. Nair et al. [26] confirmed that artemisinin was not the only anti-SARS-CoV-2 compound in the extract by showing that *A. annua* L. extracts with the lowest artemisinin content had greater antiviral efficacy. In addition, anti-SARS-CoV-2 activity was reported for aqueous extracts of Artemisia afra, an Artemisia species that does not contain artemisinin [26]. This led us to conclude that other potential anti-SARS-CoV-2 phytochemicals besides artemisinin should be investigated. Of all samples tested in this study, the arteannuin B fraction showed the highest anti-SARS-CoV-2 potential (EC_50_ of 38.1 µg/mL), with 100% inhibition of viral replication at 100 µg/mL and effective inhibition of amplification of both the N and RdRp genes (Figure 6). The arteannuin B fraction was also highly active at 50 µg/mL (*p* < 0.0001) and inhibited the amplification of SARS-CoV-2 N and RdRp genes by 84% (Figure 6). On the other hand, artemisinin did not show significant antiviral activity at the concentrations tested (EC_50_ >100 µg/mL), which could be due to the fact that higher doses are required to achieve a more pronounced effect (Figure 6). This observation is consistent with previous reports suggesting that the efficacy of artemisinin in antiviral applications may be limited compared to other *A. annua* L. compounds [22, 29]. The observed antiviral effect of the tested extracts, fractions, and compounds of *A. annua* L. was most likely not caused by DMSO, which did not independently reduce virus replication (*p* > 0.05). It is important to emphasize that an antiviral effect of the arteannuin B fraction is observed when it is used in concentrations that do not affect cell viability (IC_50_ >200 µg/mL). Although arteannuin B is reported to have anti-SARS-CoV-2 activity *in vitro* [27–30], a direct comparison of EC_50_ values is difficult due to differences in the viral strain, experimental procedures, and methods of evaluating antiviral activity in these studies compared to ours. Considering the reported broad-spectrum antiviral potential of artemisinins, several experiments have investigated the antiviral effect of artemisinins on SARS-CoV-2 with results similar to ours. Cao et al. [29] investigated the anti-SARS-CoV-2 activities of nine artemisinin-related compounds against the SARS-CoV-2 strain nCoV-2019BetaCoV/Wuhan/WIV04/2019 *in vitro* and reported that arteannuin B showed the highest anti-SARS-CoV-2 potential with an EC_50_ of 10.28 ± 1.12 µM. The semi-synthetic *A. annua* L. derivatives artesunate and dihydroartemisinin showed similar EC_50_ values of 12.98 ± 5.30 µM and 13.31 ± 1.24 µM, respectively [29].

Cao et al. also provided more direct evidence for the inhibitory effect of arteannuin B by performing an immunofluorescence assay (IFA). Viral nucleoprotein (NP) expression was completely inhibited when arteannuin B was added at a concentration of 25 µM, which was consistent with the viral yield based on qRT-PCR analysis [29]. Similar results were reported by Hu et al. [28], who used the SARS-CoV-2 isolate WIV04, with the accession number MN996528, to test the antiviral effect of certain drugs. According to Hu et al. [28], artesunate (EC_50_ ═ 16.24 µM, CC_50_ ═ 127.3 µM, SI ═ 7.84), arteannuin B (EC_50_ ═ 12.03 µM, CC_50_ ═ 116.9 µM, SI ═ 9.72), echinatin (EC_50_ ═ 7.862 µM, CC_50_ ═ 120.1 µM, SI ═ 15.27), licochalcone B (EC_50_ ═ 15.53 µM, CC_50_ ═ 106.5 µM, SI ═ 6.86), and andrographolide (EC_50_ ═ 11.12 µM, CC_50_ ═ 95.73 µM, SI ═ 8.61) all showed excellent anti-SARS-CoV-2 activity. Scopoletin, arteannuin B, and artemisinic acid (individual fractions isolated from *A. annua* L.) exerted considerable virucidal and antiviral activity *in vitro*, as confirmed by qRT-PCR, from a concentration of 50 µg/mL [27], which is consistent with our data for the arteannuin B fraction. In contrast, Cao et al. [29] reported no significant anti-SARS-CoV-2 activity for artemisinic acid. Baggieri et al. [27] presented Surface Plasmon Resonance (SPR) data and showed that the inhibition of viral infection was due to the interaction of these compounds, including arteannuin B, with 3CLpro, suggesting that the main interaction of the compounds might interfere with viral pathways during the insertion and replication process. A limitation of the study by Baggieri et al. [27] is that they only tested the antiviral activity of the individual fractions of *A. annua* L. scopoletin, arteannuin B, and artemisinic acid against the wild-type strain of SARS-CoV-2 circulating in Italy in 2020. Arteannuin B hindered the activity of the SARS-CoV-2 main protease (nonstructural protein, NSP5), a cysteine protease, through time-dependent inhibition, as reported by Varela et al. [30]. The active site cysteine residue of NSP5 (cysteine-145) formed a covalent bond with arteannuin B, as determined by mass spectrometry. These results enhance our understanding of how *A. annua* L. and its bioactive secondary metabolites possess antiviral activity. The main protease of SARS-CoV-2 is the most promising drug target against coronaviruses due to its essential role in virus replication. With newly emerging variants, there is a concern that mutations in the main protease may alter the structural and functional properties of the protease and subsequently the potency of existing and potential antivirals. Chen et al. [68] observed maintained potency of nirmatrelvir against the variants of the main protease, suggesting that the main protease remains an excellent antiviral target as the virus evolves.

An important contribution of our study is the demonstration of the antiviral activity of arteannuin B against the Alpha variant of SARS-CoV-2, which is known to have increased infectivity and transmissibility, as confirmed by genome analysis. The results of the study are relevant and significant because, unlike previous studies, they concern the transmissible and infectious strain of the virus. The question remains whether arteannuin B retains its efficacy against other strains. Genome analysis revealed the following mutations in the isolate hCoV-19/Bosnia and Herzegovina/VFS-UNSA-LMGFI031/2021, which are also found in other virus variants: (i) nsp6: Δ106-108: Alpha, Beta, Gamma, Eta, Iota, Lambda, and Omicron variants each contain the same deletion of amino acids 106–108 in non-structural protein 6, a component of the membrane-tethered replication complex of SARS-CoV-2; (ii) Spike Δ69-70: NTD deletions at positions 69 and 70 have been reported in several variants, including Omicron BA.1, BA.4, and BA.5, and are associated with increased viral replication; (iii) Spike Δ144: NTD deletions between positions 141–146 occur in the Alpha and Omicron BA.1 and BA.2 variants; (iv) Spike N501Y: N501Y is a mutation at the ACE2-binding site that occurs in the Alpha, Beta, Gamma, and each of the Omicron variants. It increases ACE2 binding; (v) Spike P681H: P681H is located proximal to the S1/S2 furin cleavage site and is present in the Alpha, Theta, and each of the Omicron variants. In this context, the importance of further experimental validation is emphasized in order to fully evaluate its potential as a broad-spectrum antiviral agent. Our results, together with those presented above [27–30] on the anti-SARS-CoV-2 activity of arteannuin B, emphasize its potential as an antiviral agent. Its unique core structure provides information for the future optimization of artemisinins as anti-SARS-CoV-2 agents. To assess its therapeutic relevance, further studies in animal models are required to understand the pharmacokinetics and pharmacodynamics. In addition, future research should focus on correlating the reported *in vitro* concentrations with achievable systemic concentrations in the clinical setting.

## Conclusion

The study makes an important contribution to the understanding of the antiviral potential of *A. annua* L. as a source of anti-SARS-CoV-2 compounds. Our study shows a clear, concentration-dependent inhibition of SARS-CoV-2 RNA replication in the supernatant of VERO E6 cells, confirming the potential of the tested ethanolic and SC-CO_2_ extracts of *A. annua* L., the artemisinin knockout fraction, and arteannuin B in attenuating viral replication.

By combining two methodological approaches one phytochemical (countercurrent chromatography) and one *in vitro* (antiviral assay on the VERO E6 cell line) arteannuin B was isolated in high purity from the crude EtOH extract of *A. annua* L. in a single chromatographic step and identified as an effective molecule against the genomically characterized Alpha SARS-CoV-2 variant. The results highlight arteannuin B as a promising antiviral agent with strong activity at concentrations of 50 µg/mL and 100 µg/mL, inhibiting the amplification of SARS-CoV-2 N and RdRp genes by up to 100%. In addition, this study is the first *in vitro* report on the antiviral effect of SC-CO_2_ extract against the Alpha variant of SARS-CoV-2.

Research also benefits from the use of the local virus isolate, providing region-specific insights that are valuable for understanding viral evolution in Bosnia and Herzegovina. The selection of the Alpha variant of SARS-CoV-2 is justified due to its epidemiological importance. Testing against Omicron sub-variants and other strains would strengthen the results. Targeted *in vitro* evaluation of artemisinin-related compounds against characterized SARS-CoV-2 variants is beneficial for assessing their antiviral spectrum and adaptability to viral mutations, which should be the focus of future research.

## Supplemental data

Supplementary data are available at the following link: https://www.bjbms.org/ojs/index.php/bjbms/article/view/12052/3851.

## Data Availability

All data obtained in this study are included in this article. The sequence data related to the isolate hCoV-19/Bosnia and Herzegovina/VFS-UNSA-LMGFI031/2021 can be accessed through the GISAID database (accession ID: EPI_ISL_1016969).
